# Sexual Satisfaction in Spanish Same-Sex Couples: Testing the Interpersonal Exchange Model of Sexual Satisfaction

**DOI:** 10.1007/s10508-025-03219-x

**Published:** 2025-09-23

**Authors:** Pablo Mangas, Inês M. Tavares, Cristóbal Calvillo, Reina Granados, Juan Carlos Sierra

**Affiliations:** 1 Mind, Brain and Behavior Research Center, University of Granada, 18011 Granada, Spain; 2 HEI-Lab: Digital Human-Environment Interaction Labs, Lusófona University, Porto, Portugal; 3 Fay W. Boozman College of Public Health, University of Arkansas for Medical Sciences, Little Rock, AR USA; 4 Department of Nursey, Health Sciences Faculty, University of Granada, Granada, Spain; 5 Facultad de Psicología, Universidad de Granada, Campus Universitario de La Cartuja, S/N, Granada, Spain

**Keywords:** Sexual satisfaction, Interpersonal Exchange Model of Sexual Satisfaction, Same-sex couples, Dyadic analysis, Sexual orientation

## Abstract

The Interpersonal Exchange Model of Sexual Satisfaction (IEMSS) is a theoretical framework that conceptualizes sexual satisfaction in couple relationships by including both sexual and non-sexual interpersonal variables. The IEMSS has not been validated dyadically in same-sex couples. This study aimed to examine the sexual satisfaction of 228 Spanish same-sex couples (116 male dyads and 112 female dyads) from the IEMSS perspective. Both men and women surveyed reported high levels of sexual and relationship satisfaction, as well as elevated scores on all IEMSS components. Men’s sexual satisfaction was explained by their own relationship satisfaction, the balance of sexual rewards and costs, and the comparison level of sexual rewards and costs. Women’s sexual satisfaction was explained by the same variables as men’s and by their partner’s balance of sexual rewards and costs and the comparison level of sexual rewards and costs. Consistent with most previous evidence, we found no effect of equality components on sexual satisfaction beyond the bivariate level. These results expand current knowledge on sexual satisfaction and its correlates in same-sex couples. Although no partner effects were found in the sample of male couples, our findings highlight the importance of assessing both partners to understand the dyadic dynamics that determine sexual satisfaction, especially in the case of women, for whom sexual satisfaction is linked to the experiences manifested by both members of the couple. The current results have implications for improving the sexual well-being and quality of life of same-sex couples.

## Introduction

Sexual satisfaction can be conceived as resulting from both individual and dyadic or interpersonal sexual experiences (Pascoal et al., 2014). One of the most widely accepted definitions of sexual satisfaction states that it is “an affective response arising from one’s subjective evaluation of the positive and negative dimensions associated with one’s sexual relationship” (Lawrance & Byers, 1995, p. 268). This definition places sexual satisfaction within an interpersonal context (Byers & Rehman, 2014). It is an essential component of people’s intimate bonding and is closely related to physical and psychological health (Laumann et al., 2006), overall well-being and quality of life (Byers & Rehman, 2014; Sánchez-Fuentes et al., 2014; Stephenson & Meston, 2011), sexual well-being (Diamond & Huebner, 2012), sexual health (Pascoal et al., 2014), relationship satisfaction (Mangas et al., 2024; McNulty et al., 2016; Vowels & Mark, 2020), and relationship stability (Yeh et al., 2006). In addition, sexual satisfaction is considered by the World Health Organization (2010) and the World Association for Sexual Health (Kismödi et al., 2017) as a sexual right.

Historically, the sexuality of people belonging to sexual orientation and gender diversities (SOGDs) has often been excluded from research (Serrano-Amaya & Ríos-González, 2019). Several authors (e.g., Andersen & Zou, 2015; Budge & Katz-Wise, 2019; Diamond & Blair, 2018; Mark et al., 2015; Pascoal et al., 2019; Totenhagen et al., 2012) have noted that research on intimate relationships has tended to focus on people with different-sex partners. Unfortunately, excluding SOGD individuals from scientific research could negatively affect these people’s physical and psychological health (Lick et al., 2013; Parra et al., 2016). There is therefore a clear need for research to be more inclusive, especially for groups that are more affected by the consequences of social prejudice (Frost, 2011). To this end, it is necessary to validate and adapt instruments and theoretical models to diverse populations, especially same-sex couples, since they present particularities that make them unique (e.g., Frost et al., 2017; Rostosky & Riggle, 2017; Umberson et al., 2018), many of which are derived from the consequences of stigma and which have been shown to negatively affect sexual satisfaction (Cohen & Byers, 2015; Vale & Bisconti, 2021).

While the relationships among LGBT+ people are increasingly visible, most studies examining sexual satisfaction have focused on heterosexual couples (Fleishman et al., 2020; Mangas & Sierra, 2025; Pascoal et al., 2019). This gap in research on sexual satisfaction in both male–male (Newcomb et al., 2021) and female–female (Holt et al., 2021) couples is evident, especially for LGBT+ older adult couples (Fleishman et al., 2020). A systematic review by Calvillo et al. (2018) revealed that sexual satisfaction in same-sex couples, as occurs in different-sex couples, is associated with personal, interpersonal, social, and ideological-cultural variables, among which some factors stand out, particularly within same-sex couples. These factors include sex and/or gender roles. With respect to sex differences in the gay population, previous evidence indicates that women present higher levels of sexual satisfaction than men do (Calvillo et al., 2020a, 2020b; Guzmán-González et al., 2019; Holmberg & Blair, 2009).

The present study takes the only theoretical model of sexual satisfaction validated in the Spanish population as a reference: the Interpersonal Exchange Model of Sexual Satisfaction (IEMSS; Lawrance & Byers, 1992, 1995). Its Spanish validation was carried out exclusively in different-sex couples by Sánchez-Fuentes and Santos-Iglesias (2016). This model is a conceptual framework developed from the perspective of social exchange theory (Thibaut & Kelley, 1959) to understand sexual satisfaction in the context of couple relationships (Byers & Rehman, 2014). The IEMSS focuses on interpersonal variables, both sexual and non-sexual, that explain sexual satisfaction independently of the effect of sociodemographic factors, such as sex or relationship length (Byers & MacNeil, 2006; Byers & Rehman, 2014; Lawrance & Byers, 1995). The strength of the IEMSS lies in the fact that it was conceived to improve prior research for several reasons: (1) it assesses sexual satisfaction on the basis of more parameters than proposals based on a single question, (2) it includes non-sexual aspects of the couple relationship, (3) it is a model that reflects the interpersonal nature of sexual satisfaction, which makes it ideal for dyadic studies, (4) it considers the role of expectations (e.g., perceived equality is contemplated), and (5) it compares partner parameters (i.e., assesses comparative levels between members). A now-classic model, the IEMSS is still used today and continues to be a theoretical reference in the study of sexual satisfaction and the factors that influence it.

According to the IEMSS theoretical framework (Lawrance & Byers, 1992, 1995), four components explain sexual satisfaction: (1) the balance of sexual rewards and costs in a sexual relationship (REW–CST); (2) how these sexual rewards and costs compare with the expected levels of rewards and costs, named comparison/relative levels of sexual rewards and costs (CL_REW_–CL_CST_); (3) the perceived equality of sexual rewards and costs between members of the couple (EQ_REW_ and EQ_CST_); and (4) the quality of the non-sexual aspects of the relationship (GMREL). Sexual rewards are exchanges experienced and interpreted as positive, pleasurable and enriching; in contrast, sexual costs are exchanges that require effort or cause pain, anxiety, or other negative feelings or emotions (Lawrance et al., 2020; Thibaut & Kelley, 1959). As proposed in the IEMSS, in the context of a couple relationship, sexual satisfaction is greater as long as (1) they experience high sexual rewards and low sexual costs, resulting in a favorable balance between the overall level of sexual rewards and the level of sexual costs; (2) this balance is favorable in comparison with one’s expectations, or one’s anticipated level of sexual rewards and costs; (3) there is a perception of greater equality between one’s own sexual rewards and costs and those of one’s partner; and (4) they value the non-sexual elements of the relationship more highly (Byers & MacNeil, 2006; Byers et al., 1998; Lawrance & Byers, 1995; Renaud et al., 1997).

In recent years, the IEMSS has been the objective of several studies. There is abundant previous evidence indicating that the perceived equality components (EQ_REW_ and EQ_CST_) have little effect on sexual satisfaction. In contrast, at the bivariate level, results for the heterosexual population (Byers et al., 1998; Lawrance & Byers, 1995; Renaud et al., 1997) accounts for the association of all components with sexual satisfaction, with more salience of EQ_REW_ in long-term couples (Lawrance & Byers, 1995), and more of EQ_CST_ in short-term dating relationships (Byers et al., 1998). The results are more diverse in terms of which sexual exchange construct contributed uniquely to the prediction of sexual satisfaction. For example, in Byers et al. (1998) only CL_REW_–CL_CST_ and EQ_CST_ contributed to sexual satisfaction, whereas in Peck et al. (2005), REW–CST, CL_REW_–CL_CST_ and EQ_REW_ contributed to sexual satisfaction. More than a decade later, studies began to implement a dyadic approach, specifically after the work of Byers and MacNeil (2006). In this study, although EQ_REW_ and EQ_CST_ were associated with sexual satisfaction at the bivariate level, the perceived equality variables were not uniquely associated with sexual satisfaction, as occurred in La France’s (2010) study, where the sexual equality variables were the least strongly related to sexual satisfaction. La France’s study was the first to include non-heterosexual individuals, although they still represented a minority (4%). Yucel and Gassanov (2010) were the first to use Actor Partner Interdependence Models (APIM) and expressed the need to address actor-partner effects. Byers and Nichols (2014), using a sample of neurodivergent individuals, reported that only GMREL and REW–CST contributed to sexual satisfaction. The first integral approximation to sexual diversities was performed by Byers and Cohen (2017), who established that all the components of the IEMSS, except for EQ_CST_, uniquely contributed to sexual satisfaction.

Additionally, studies have also examined other phenomena (e.g., sexual functioning, sexual double standard) under the framework of the IEMSS, such as Stephenson and Meston (2011) or Álvarez-Muelas et al. (2023). Other studies have employed qualitative methodologies, such as Raisi et al. (2015) sampling Iranian married women, or Santos-Iglesias and Byers (2021) sampling older adults. Fallis et al. (2016) reaffirmed the connection between sexual satisfaction and relationship satisfaction using a longitudinal design, similar to that employed by Péloquin et al. (2024), whose results suggested that attachment insecurities were associated with sexual exchanges over time. Additionally, Rosen et al. (2017) presented the IEMSS components as capable of capturing differences between clinical and non-clinical samples of women with provoked vestibulodynia.

The evidence found so far in the Spanish population is similar to that of studies conducted in other contexts. The study of Sánchez-Fuentes et al. (2015) indicated that EQ_REW_ and EQ_CST_ made the smallest contribution to sexual satisfaction out of all the IEMSS components, although in Sánchez-Fuentes and Santos-Iglesias (2016), a positive actor effect of EQ_CST_ in heterosexual women was observed. With respect to non-heterosexual populations, Calvillo et al. (2020a) have noted the low effect of the perceived equality components, which is the reason why these components were excluded in their validation of the model in gay individuals in a romantic relationship.

From a contextual perspective, the IEMSS has been validated in different-sex couples from diverse countries, including Spain (Sánchez-Fuentes & Santos-Iglesias, 2016), the United States (Peck et al., 2005), Canada (Byers et al., 1998), and China (Renaud et al., 1997). In terms of relationship length, although the model was developed for long-term relationships and appears ideally suited for this type of relationships (Byers & MacNeil, 2006; Lawrance & Byers, 1995), Byers et al. (1998) expanded the validity of the model to young and short-term couples as well. In the present study, we decided to include couples at different stages on the basis of the authors’ statement that “the model was shown to work equally well for individuals new and less new to their relationship” (Byers et al., 1998, p. 266), a similar approach to that considered by recent evidence sampling sexual diversities (Byers & Cohen, 2017; Calvillo et al., 2020a, 2020b). Overall, the current evidence suggests that the previous findings are not moderated by the duration of the couple relationship (e.g., Byers & Cohen, 2017; Byers & Nichols, 2014).

Although the IEMSS is conceptualized in interpersonal terms, most studies employ this theoretical model—or are based on it—to assess the sexual satisfaction of individuals in a romantic relationship, either in different-sex relationships (Byers et al., 1998; Lawrance & Byers, 1995; Renaud et al., 1997), same-sex relationships (Byers & Cohen, 2017; Calvillo et al., 2020a, 2020b), or with mixed samples comprising both different-sex and same-sex couples (Byers & Nichols, 2014; Li & Samp, 2022; Santos-Iglesias & Byers, 2021). However, few studies focus on the couple as the unit of analysis (Byers & MacNeil, 2006; Rosen et al., 2017; Sánchez-Fuentes & Santos-Iglesias, 2016; Yucel & Gassanov, 2010). Among all dyadic studies conducted to date, no study has included a sample of same-sex couples. Freihart et al. (2020), who prioritize dyadic strategies, criticized the lack of sufficient research on sexual satisfaction within sexual diversity couples.

### The Current Study

In the present study, we aimed to expand the current knowledge by overcoming two previous limitations: the exclusive inclusion of couples (and not individuals in a romantic relationship) and, specifically, same-sex couples. Therefore, this is the first study that uses APIM to validate the IEMSS in same-sex dyads to show the contribution of each partner’s sexual satisfaction components to their own (actor effects) and to their partner’s (partner effects) sexual satisfaction.

In this work, in addition to the IEMSS (Lawrance & Byers, 1992, 1995), we relied on the “Gender-as-Relational” (GAR) approach (Thomeer et al., 2020; Umberson et al., 2018). This framework shows that bonds are highly conditioned by gender and that women and men experience their relationships differently (Thomeer et al., 2013). The GAR lens suggests different manifestations of intimacy according to the gender configuration of the couple: men with men, men with women, women with men, and women with women (Thomeer et al., 2020), a finding that has already been observed in recent studies employing this paradigm, sampling couples with partners from sexual diversity populations (Mangas et al., 2024, 2025; Pérez-Amorós et al., 2024). For example, Blair et al. (2017) argued that relationship configuration is a more influential factor than self-identified sexual orientation is, which helps explain how sexual activity varies across relationships. In addition to sexual satisfaction (e.g., Calvillo et al., 2020a, 2020b), other psychosexual variables also vary depending on the gender configuration of the relationship, such as emotional intimacy (Guzmán-González et al., 2021; Šević et al., 2016) or orgasm (Blair et al., 2017).

**Fig. 1:**
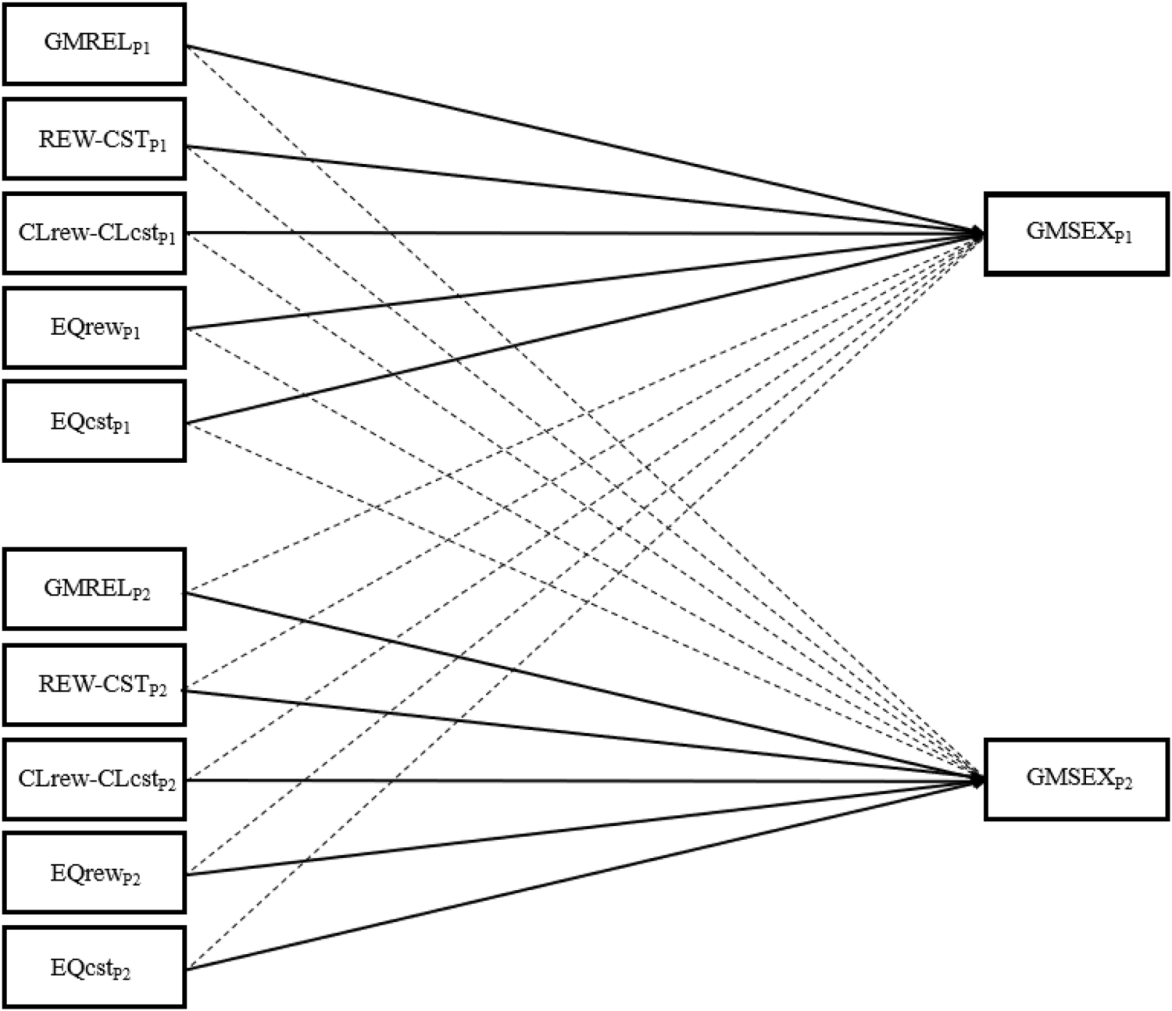
Full model of the Interpersonal Exchange Model of Sexual Satisfaction in couples. *Note*. GMSEX, Global Measure of Sexual Satisfaction; GMREL, Global Measure of Relationship Satisfaction; REW–CST, balance of sexual rewards to costs; CL_REW_–CL_CST_, comparison level of sexual rewards to costs; EQ_REW_, equality of sexual rewards; EQ_CST_, equality of sexual costs; P1/P2, partner 1/partner 2. The hypothesized model is valid for both male and female couples. Assignment to P1 and P2 was randomized. Solid lines indicate actor effects and dotted lines indicate partner effects

Here, we tested the validity of the IEMSS (Lawrance & Byers, 1992, 1995) in male and female same-sex couples separately. Despite the absence of research that has dyadically studied the sexual satisfaction of same-sex couples under this consolidated theoretical model, and on the basis of studies conducted in different-sex dyads (e.g., Byers & MacNeil, 2006; Lawrance & Byers, 1995; Sánchez-Fuentes & Santos-Iglesias, 2016) and on findings in people with same-sex partners (e.g., Byers & Cohen, 2017; Calvillo et al., 2020a), we hypothesized that: (1) there will be actor effects of the different components of the IEMSS (i.e., GMREL, REW–CST, CL_REW_–CL_CST_, EQ_REW_, and EQ_CST_) on the sexual satisfaction of male and female same-sex couples (H1), and (2) partner effects will be observed in the explanation of sexual satisfaction in the case of female same-sex couples (H2). Being the first study of this nature, we consider it premature to predict which specific components are expected to show both actor and partner effects given the sample used and the analytical strategy selected. All possible effects on the IEMSS are illustrated in Fig. 1.

## Method

### Participants

The following inclusion criteria were considered: (1) having Spanish nationality; (2) being at least 18 years of age; (3) being cisgender; (4) having a relationship with a duration of at least 3 months with someone of the same sex and cisgender; and (5) the partner, who must also meet the inclusion criteria, having participated in the study. A non-probabilistic sampling was used to recruit 228 same-sex couples from the Spanish population, which were divided into two subsamples: (1) male–male dyads (*n* = 116) and (2) female–female dyads (*n* = 112), with an age range of 18–58 years (*M* = 28.15; *SD* = 7.38) and a mean length of the relationship of 44.69 months (*SD* = 47.04). Most participants had university studies (78.8% of men and 86.1% of women). The mean age of the first sexual relationship in men was 16.61 years, and they had an average of 44.7 lifetime sexual partners. Among women, the mean age of the first sexual relationship was 17.02 years, and they had an average of 8.22 lifetime sexual partners. Moreover, 84.8% of men and 93.3% of women were in exclusive relationships, and 89.2% of men and 55.4% of women had sexual relationships exclusively with people of the same sex. The sociodemographic characteristics of the participants are organized by sex in Table 1. If data were missing in the scales or participants failed to comply with the inclusion criteria, the participant and their partner were excluded from the study.

**Table 1:** Sociodemographic characteristics of the participants

	Men (*n* = 232) (116 dyads)			Women (*n* = 224) (112 dyads)		
	Range	*M* (*SD*)	*Mₑ*	Range	*M* (*SD*)	*Mₑ*
Age (years)	18–52	29.67 (7.36)	28	18–58	26.57 (7.08)	24
	*n* (%)		*n* (%)			
Education level						
Primary education	2 (0.9)		1 (0.4)			
Secondary education	47 (20.3)		30 (13.5)			
University degree	182 (78.8)		192 (86.1)			
	*M* (*SD*)		*M* (*SD*)			
Age of first sexual relationship (in years)	16.61 (3.10)		17.02 (2.64)			
	*n* (%)		*n* (%)			
Relationship exclusivity						
Exclusive	196 (84.8)		208 (93.3)			
Non-exclusive	35 (15.2)		15 (6.7)			
Cohabitation						
Yes	140 (60.3)		92 (41.3)			
No	92 (39.7)		131 (58.7)			
	*M* (*SD*)	Range	*Mₑ*	*M* (*SD*)	Range	*Mₑ*
Relationship length (in months)	51.90 (46.94)	3–248	36	37.42 (46.09)	3–305	24
	*Mₑ*	*M* (*SD*)	*Mₑ*	*M* (*SD*)		
Number of lifetime sexual partners	15	44.70 (107.36)	5	8.22 (10.07)		
	*n* (%)		*n* (%)			
Type of sexual practices						
Mostly heterosexual but more than slightly homosexual	0 (0)		1 (0.4)			
Equally heterosexual and homosexual	12 (5.2)		64 (28.6)			
Mostly homosexual but more than slightly heterosexual	1 (0.4)		7 (3.1)			
Mostly homosexual, only slightly heterosexual	12 (5.2)		28 (12.5)			
Exclusively homosexual	207 (89.2)		124 (55.4)			

*Mₑ*, median; *M*, mean, *SD*, standard deviation

### Measures

#### Sociodemographic and Sexual History Questionnaire

We collected sociodemographic information about sex, gender, age, nationality, education level, type of sexual practices, relationship data (i.e., sex, gender, and age of the partner, relationship length, exclusivity/non-exclusivity, cohabitation, and sexual relationships with the partner), and sexual history variables (i.e., age of first sexual relationship and number of lifetime sexual partners).

#### Spanish Version of the Global Measure of Sexual Satisfaction (GMSEX; Sánchez-Fuentes et al., 2015***)***

The GMSEX is composed of five 7-point bipolar scales (Very bad/Very good; Very unpleasant/Very pleasant; Very negative/Very positive; Very unsatisfying/Very satisfying; Worthless/Very valuable), which allow the assessment of sexual relationships with the partner. The scores ranged from 5 to 35 points, such that higher scores represented greater sexual satisfaction in the context of the couple relationship. Both in its original version (Byers & MacNeil, 2006; Lawrance & Byers, 1995) and in the Spanish adaptation (Calvillo et al., 2020a; Sánchez-Fuentes et al., 2015), high values of internal consistency were reported, as well as adequate evidence of validity. In this study, Cronbach’s alpha was 0.88 for both the male and female samples.

#### Spanish Version of the Global Measure of Relationship Satisfaction (GMREL; Sánchez-Fuentes et al., 2015***)***

This measure is similar to GMSEX but assesses global satisfaction with the couple relationship. Similarly, scores ranged from 5 to 35, such that higher scores represented greater satisfaction with the couple relationship. It had good reliability and validity indicators, both in its original version (Byers & MacNeil, 2006; Lawrance & Byers, 1995) and in its validation in the Spanish population (Calvillo et al., 2020a; Sánchez-Fuentes et al., 2015). In the present work, Cronbach*’*s alpha was 0.87 for the sample of men and 0.90 for the sample of women.

#### ***Spanish Version of the Exchanges Questionnaire (EQ; ***Sánchez-Fuentes et al., 2015***)***

The EQ consists of six items that assess four of the components of the IEMSS: overall balance of sexual rewards and costs (REW–CST), comparison of perceived levels of sexual rewards and costs with the expected levels of sexual rewards and costs (CL_REW_–CL_CST_), perceived equality of sexual rewards between members (EQ_REW_), and perceived equality of sexual costs between members (EQ_CST_). The first three items focus on sexual rewards, such that item 1 assesses the overall level of sexual rewards received in sexual relationships with a partner in the last three months, using a 9-point Likert-type scale ranging from (1) *Not at all rewarding* to (9) *Extremely rewarding*. Item 2 assesses the level of sexual rewards compared with the level of expected sexual rewards on a 9-point Likert scale ranging from (1) *Much less rewarding in comparison* to (9) *Much more rewarding in comparison*. Item 3 assesses the level of one’s rewards in comparison with the level of rewards received by the partner on a 9-point Likert scale ranging from (1) *My rewards are much higher* to (9) P*artner’s rewards are much higher*. Parallel items 4, 5, and 6 are similarly formulated to assess sexual costs.

The overall balance of rewards and costs (REW–CST) was obtained by subtracting the scores of item 4 from item 1. The comparison level of sexual rewards and costs (CL_REW_–CL_CST_) was obtained by subtracting the scores of item 5 from item 2. For both components, the scores ranged between -8 and + 8, such that higher scores represented a more favorable balance of sexual rewards to sexual costs. To obtain the components of the Perceived equality of sexual rewards (EQ_REW_) and sexual costs (EQ_CST_), the scores of items 3 and 6 were recoded so that a score of 4 was assigned to the midpoint of the scale (i.e., perfect equality) and 0 to both extremes. Thus, higher scores indicate greater perceived equality of sexual rewards/costs between partners. The original version (Byers & MacNeil, 2006; Lawrance & Byers, 1995), its adaptation to Spanish (Sánchez-Fuentes et al., 2015), and its validation in Hispanic individuals in a romantic relationship (Calvillo et al., 2020a) showed optimal psychometric properties.

### Procedure

A battery of online questionnaires was used, a common procedure for assessing sexual behaviors in non-heterosexual populations (Calvillo et al., 2020a; Frankis et al., 2018). Open software LimeSurvey®, located on the servers of the University of Granada, was used to disseminate the survey through social media and specialized communication channels (e.g., LGBT+ groups and associations) among adults from all over Spain. Data collection was carried out between March and April 2022.

All the participants were informed of the purpose and voluntary nature of the study, the characteristics of the evaluation, and the implications of their participation. Anonymity, data protection, and confidentiality of their responses were assured throughout. No personal information beyond their initials and birth years (to identify the dyads) was required at any time. All the participants voluntarily agreed to take part in the study by providing informed consent. The approximate time to complete the questionnaire was about 15 min.

To avoid fraudulent or bot-generated responses, a CAPTCHA based on a random arithmetic operation was included at the beginning of the survey. The data were thoroughly reviewed to eliminate cases with inusual response patterns. At the end of the study, we ensured that it met adequate quality indices by reviewing the STROBE Statements (von Elm et al., 2007).

Although the battery of questionnaires was to be filled out individually, to allow for partner participation, the couple link was provided so that it could be easily sent. To link the responses of both members of the couple and guarantee anonymity, we requested the following information to unify the codes and generate a common alphanumeric code: (1) initials of the first and last names of the youngest member of the couple, (2) initials of the first and last names of the oldest member of the couple, (3) the last two digits of the year of birth of the youngest member of the couple, and (4) the last two digits of the year of birth of the oldest member of the couple, following the recommendations of Mitchell et al. (2020).

### Data Analysis

The necessary sample size calculation was conducted using the APIMPowerR software (Ackerman & Kenny, 2016). It was estimated that a sample size of 90 dyads per group would be required to achieve adequate statistical power to detect both actor and partner effects (α = 0.05, desired power = 0.95, actor and partner size = 0.25). Couples participating in the study were analyzed separately according to sex because they were considered contextually different, following the GAR approach (Thomeer et al., 2020; Umberson et al., 2018). When the full sample was tested and sex was used as a moderator, the results were maintained.

The intercorrelations between the variables were calculated, first within individuals, as well as between partners, separately for men and women. Actor-Partner Interdependence Models (APIM; Kenny et al., 2006) using multilevel modeling, where partners were nested within couples, were used to test the IEMSS models. APIM simultaneously estimates actor effects (association between predictors and own outcome) and partner effects (association between partner predictors and own outcome). To check that the data were suitable for dyadic analyses, we first examined the interdependence of the scores of both partners via Pearson correlations.

The dyads included in the study were considered indistinguishable following the recommendations of Cook and Kenny (2005). In same-sex couples, there is no systematic or meaningful ways to distinguish between members of a dyad (in this case, both are actors and partners), resulting in only one actor effect and one partner effect per association (Olsen & Kenny, 2006). Therefore, each member of the pair was randomly assigned as “Partner 1 (P1)” or “Partner 2 (P2)”. GMSEX was conceived as the outcome variable, and the remaining components of the IEMSS model were considered predictor variables (i.e., GMREL, REW–CST, CL_REW_–CL_CST_, EQ_REW_, and EQ_CST_). IBM® SPSS® Statistics v.22 software was used for statistical data analysis. We also used the ItoP and ItoD applications for data restructuring, developed by Ledermann and Kenny (2015).

## Results

In general, the results obtained are congruent with previous research in different-sex couples and individuals in same-sex relationships, supporting the validity of the IEMSS in Spanish same-sex couples. We first describe the results found at the bivariate level and then the components that contributed uniquely to the prediction of sexual satisfaction.

As shown in Table 2, in the case of men, significant positive within-individual correlations were found between all the components (except for EQ_REW_) and GMSEX. With respect to the correlations between partners, a similar pattern is observed. In relation to the correlations between predictors, in general terms, significant positive correlations were observed between all the components, except for EQ_REW_, and partially for EQ_CST_. For women, positive and significant within-individual correlations were obtained between all the components and GMSEX, except for EQ_REW_. For the correlations between partners, significant positive correlations were obtained between GMREL, REW–CST, and CL_REW_–CL_CST_ and GMSEX. With respect to the correlations between predictors, significant and positive correlations were observed between all the variables except for the equality components (especially EQ_REW_, which only correlated significantly with EQ_CST_ in the within-individual analysis).

**Table 2:** Correlations among variables and descriptive statistics of the Interpersonal Exchange Model of Sexual Satisfaction in same-sex couples

	Within individuals (full sample of men and full sample of women)						Between partners (men_P1_ *×* men_P2_)						Between partners (women_P1_ *×* women_P2_)					
	1	2	3	4	5	6	1	2	3	4	5	6	1	2	3	4	5	6
1. GMSEX	–	.67***	.54***	.54***	.08	.15*	.45***						.32***					
2. GMREL	.70***	–	.37***	.43***	.01	.08	.33***	.45***					.23***	.25***				
3. REW–CST	.67***	.49***	–	.71***	.04	.14*	.37***	.22**	.45***				.34***	.29***	.61***			
4. CL_REW_–CL_CST_	.54***	.32***	.67***	–	.03	.12	.34***	.21**	.40***	.42***			.40***	.28***	.52***	.42***		
5. EQ_REW_	.10	.03	.08	−.03	–	.54***	.06	−0.03	.02	.06	− .10		.04	.06	−0.05	−.02	.05	
6. EQ_CST_	.17**	.08	.17**	.14*	.32***	–	.23**	.12	.25***	.21**	.02	.18**	.06	.06	.07	.09	.08	.07
	Men (*n* = 232)						Men_P1_ (*n* = 116)						Women_P1_ (*n* = 112)					
*M* (*SD*)	30.56 (4.47)	31.59 (4.11)	4.5 (3.24)	3.66 (3.16)	2.45 (1.44)	3 (1.19)	30.55 (4.58)	31.43 (4.26)	4.58 (3.29)	3.87 (3.27)	2.62 (1.46)	2.99 (1.18)	31.70 (4.54)	32.61 (3.78)	4.93 (3.10)	4.33 (3.20)	2.51 (1.50)	2.96 (1.29)
	Women (*n* = 224)						Men_P2_ (*n* = 116)						Women_P2_ (*n* = 112)					
*M* (*SD*)	31.65 (4.85)	32.49 (3.85)	5.07 (3.09)	4.54 (3.22)	2.64 (1.47)	3.09 (1.28)	30.56 (4.38)	31.75 (3.97)	4.41 (3.20)	3.45 (3.05)	2.28 (1.41)	3 (1.21)	31.61 (5.16)	32.38 (3.93)	5.21 (3.10)	4.74 (3.24)	2.77 (1.43)	3.22 (1.26)
Range	5–35	5–35	− 8 + 8	− 8 + 8	0–4	0–4	5–35	5–35	− 8 + 8	− 8 + 8	0–4	0–4	5–35	5–35	− 8 + 8	− 8 + 8	0–4	0–4

P1, Partner 1; P2, Partner 2: GMSEX, Global Measure of Sexual Satisfaction; GMREL, Global Measure of Relationship Satisfaction; REW–CST, Balance of sexual rewards to costs; CL_REW_–CL_CST_, Comparison level of sexual rewards to costs; EQ_REW_, Equality of sexual rewards; EQ_CST_, Equality of sexual costs. The first sector represents within-individual correlations, where values below the diagonal are based on men’s scores and values above the diagonal are based on women’s scores. The second and third sectors represent correlations between couple partners, first in male couples and next in female couples. *M*, Mean; *SD*, Standard deviation.

*p < .05, **p < .01, ***p < .001

The results obtained in the correlation analyses indicate the presence of interdependence in the data and that they are appropriate for dyadic analyses (Cook & Kenny, 2005; Kenny et al., 2006). Table 2 also shows that both men and women reported high levels of sexual and relationship satisfaction. All components of the IEMSS also denote high levels of sexual satisfaction. The Akaike’s information criterion (AIC) and Schwarz’s/Bayesian information criterion (BIC) were used to determine the fit of the APIM models. These values were used to compare the quality of the APIM models in comparison with alternative models, selecting the model with the lowest AIC and BIC for the best fit to the data, following recommendations by McCoach et al. (2022). When the final models were compared, the male couple model had a slightly better balance between fit and complexity than the female model.

**Fig. 2:**
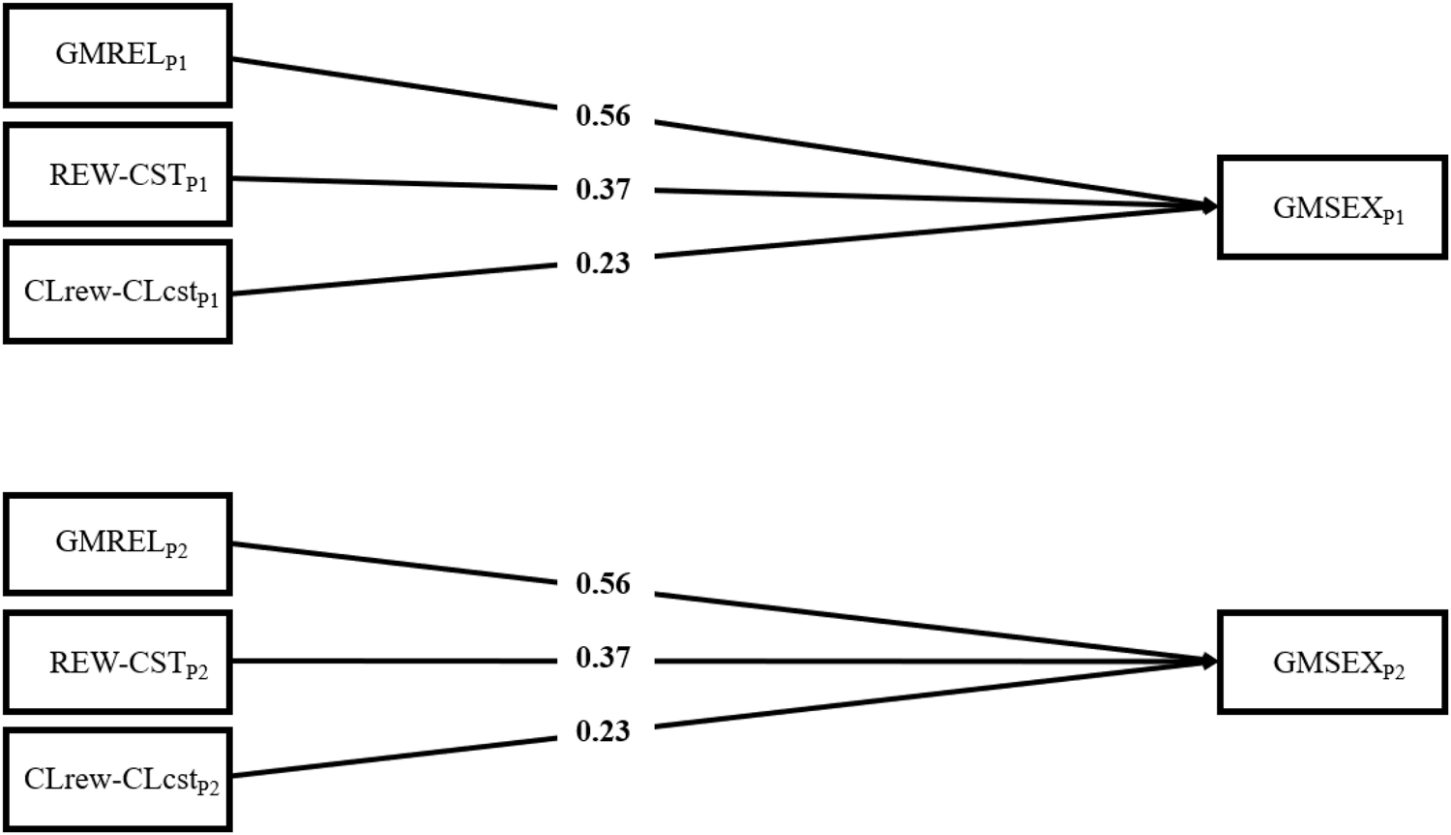
Path diagram of the Interpersonal Exchange Model of Sexual Satisfaction in male couples. Note. GMSEX, Global Measure of Sexual Satisfaction; GMREL, Global Measure of Relationship Satisfaction; REW–CST, Balance of sexual rewards to costs; CL_REW_–CL_CST_, Comparison level of sexual rewards to costs. Assignment to P1 and P2 was randomized. Solid lines indicate actor effects. Only significant paths are shown. As dyads were specified as indistinguishable, there was only one actor effect, hence the results of the upper and lower halves were replicated

In couples of men, APIM results (AIC = 1127.18; BIC = 1133.98) indicated three significant actor effects: GMREL (β = 0.56, *SE* = 0.05, *p* < 0.001), REW–CST (β = 0.37, *SE* = 0.08, *p* < 0.001), and CL_REW_–CL_CST_ (β = 0.23, *SE* = 0.07, *p* = 0.002). Higher scores on GMREL, REW–CST, and CL_REW_–CL_CST_ were associated with greater sexual satisfaction (Fig. 2). No significant partner effect was observed, indicating that none of the IEMSS components were significantly linked with partner sexual satisfaction.

**Fig. 3:**
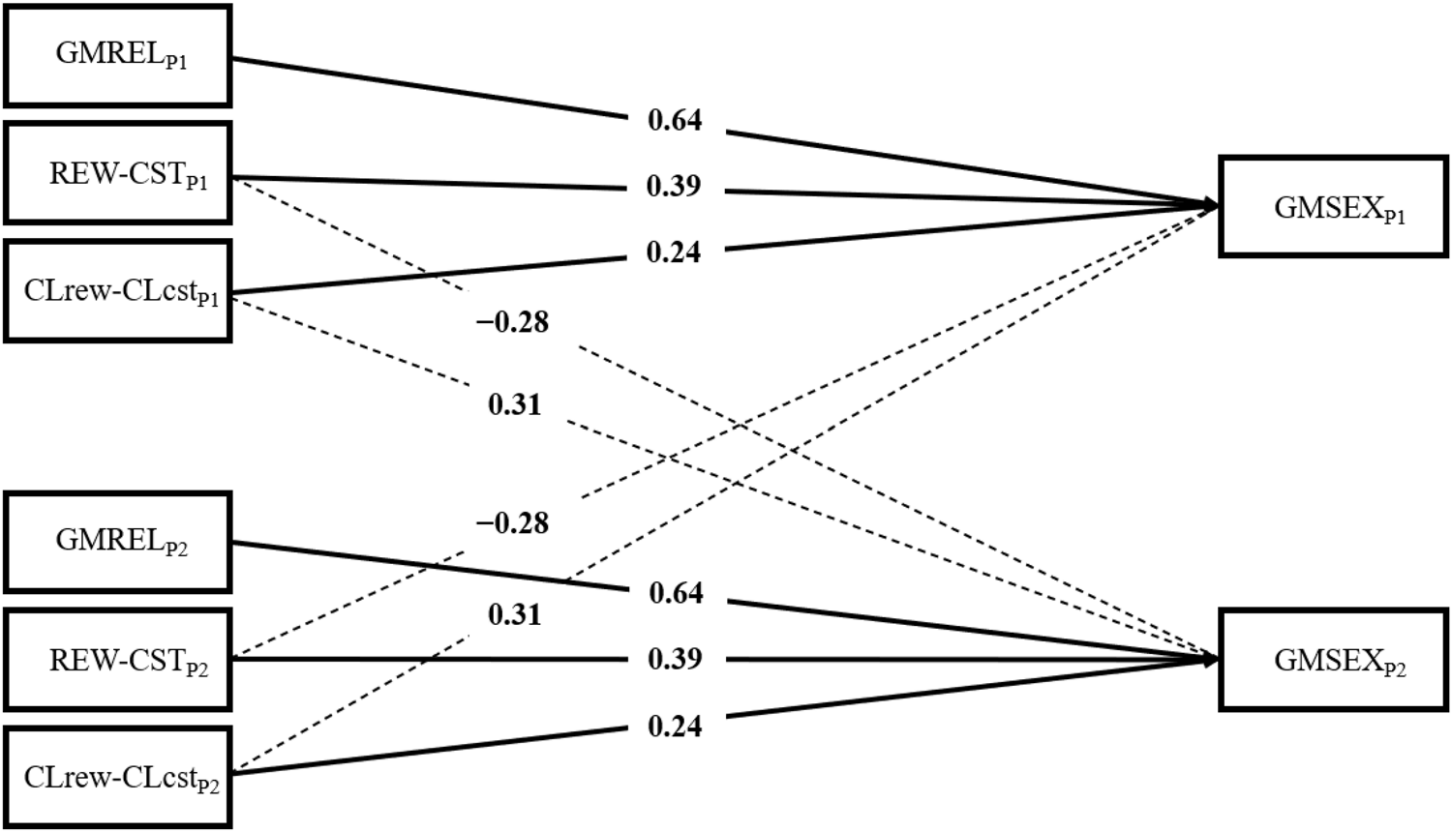
Path diagram of the Interpersonal Exchange Model of Sexual Satisfaction in female couples. *Note*. GMSEX, Global Measure of Sexual Satisfaction; GMREL, Global Measure of Relationship Satisfaction; REW–CST, Balance of sexual rewards to costs; CL_REW_–CL_CST_, Comparison level of sexual rewards to costs. Assignment to P1 and P2 was randomized. Solid lines indicate actor effects and dotted lines partner effects. Only significant paths are shown. As dyads were specified as indistinguishable, there was only one actor and one partner effect; hence, the results of the upper and lower halves were replicated

In couples of women (AIC = 1175.65; BIC = 1182.38), significant actor effects were found for the same three variables as in men: GMREL (β = 0.64, *SE* = 0.06, *p* < 0.001), REW–CST (β = 0.39, *SE* = 0.11, *p* < 0.001), and CL_REW_–CL_CST_ (β = 0.24, *SE* = 0.10, *p* = 0.015). In addition, unlike in the male dyads, we observed two partner effects: REW–CST (β = -0.28, *SE* = 0.11, *p* = 0.01) and CL_REW_–CL_CST_ (β = 0.31, *SE* = 0.10, *p* = 0.002). Similarly to men, women reported higher levels of sexual satisfaction when satisfaction with their couple relationship was high, when rewards exceeded sexual costs, and when the comparative level between sexual rewards and costs exceeded those expected. Meanwhile, unlike men, the greater the difference between partners’ sexual rewards and costs (REW–CST), the lower degree of sexual satisfaction experienced by women. Moreover, the greater the comparative level between perceived sexual rewards and costs in their partners (CL_REW_–CL_CST_), the greater the degree of sexual satisfaction reported (Fig. 3). As in the case of men, the EQ_REW_ and EQ_CST_ components had no significant effect on self or partner sexual satisfaction.

## Discussion

Our findings support the validity of the IEMSS (Lawrance & Byers, 1992, 1995) in Spanish same-sex couples. This study extends the results of previous research, especially in Spanish different-sex couples (Sánchez-Fuentes & Santos-Iglesias, 2016), showing the IEMSS as a theoretical framework from which to also study same-sex couples, providing a solid conceptual basis for future research investigating sexual satisfaction and its components in more diverse relational configurations.

Overall, the current results show that Spanish same-sex couples are satisfied with their sexual relationships and couple relationships, and have high scores on all IEMSS components. These findings are consistent with results from previous studies (e.g., Byers & Cohen, 2017; Byers & MacNeil, 2006; Calvillo et al., 2020a; Sánchez-Fuentes & Santos-Iglesias, 2016).

Although the novel contribution of our study was the dyadic approach, the results revealed that, at the bivariate level, most of the components of the IEMSS (except EQ_REW_) were positively associated with individual reports of sexual satisfaction in both men and women, as would be expected given previous evidence derived from the use of this theoretical model. The between-partner analysis also reflects an association between the variables of the model and sexual satisfaction, except for the equality components (where only the effect of EQ_CST_ on sexual satisfaction is observed in male partners). Given the nature of the sample participating in this study, mostly young adults, this is consistent with the evidence presenting EQ_CST_ as more salient in short-term couple relationships (Byers et al., 1998). Except for EQ_REW_ and EQ_CST_, whose effects on sexual satisfaction seem to be less robust, we found that the different components of the model act in a similar way to previous studies. This analysis also supports the interdependence of the data necessary for dyadic analyses (Cook & Kenny, 2005; Kenny et al., 2006).

Regarding the first hypothesis, in the results of the APIMs (Cook & Kenny, 2005; Kenny et al., 2006), we observed three significant actor effects in men: relationship satisfaction (GMREL), overall balance of sexual rewards and costs (REW–CST), and comparison levels of sexual rewards and costs (CL_REW_–CL_CST_), with them reporting higher levels of sexual satisfaction when satisfaction with their couple relationship is high, when their sexual rewards exceed costs, and when the comparative level between perceived sexual rewards and costs exceeds the expected one. Our results showed that the two equality components, EQ_REW_ and EQ_CST_, were not uniquely linked to sexual satisfaction, which is consistent with previous studies with heterosexual (Byers & MacNeil, 2006; Byers et al., 1998; Lawrance & Byers, 1995; Renaud et al., 1997; Sánchez-Fuentes & Santos-Iglesias, 2016; Sánchez-Fuentes et al., 2015) and gay samples (Calvillo et al., 2020a). Together, these studies suggest that both components of equality are hardly relevant for people with high levels of sexual satisfaction—as is the case of our study—and play a more significant role in sexually unsatisfied people (Byers & MacNeil, 2006). However, as we have previously noted, EQ_CST_ is associated with sexual satisfaction at the bivariate level, highlighting the importance of this component in understanding sexual satisfaction, in line with previous studies of a similar nature (e.g., Byers et al., 1998; Calvillo et al., 2020a; Sánchez-Fuentes & Santos-Iglesias, 2016).

Regarding the second hypothesis, in male couples no significant partner effect was found, indicating that the sexual satisfaction of each partner depends exclusively on their components and not on those of their partner. Possible explanations for this finding may be the feeling of competitiveness between men present in same-sex couples (Guzmán-González et al., 2016), the implicit and restrictive male gender norms (Mohr et al., 2013; O’Neil, 2008), or the emotional disconnection with their partners (Green & Mitchell, 2015). Socially, men are encouraged to satisfy their own sexual needs and desires (Miller & Byers, 2004) and are labeled as sexually selfish (Wetzel & Sanchez, 2022), which could explain the low effect of their partners’ equality components on their experience of sexual satisfaction. Although partner experience does not uniquely contribute to the prediction of sexual satisfaction, this contrasts with the findings at the bivariate level, where all components except EQ_REW_ were associated with sexual satisfaction in the between-partner analysis. This discrepancy is common in the study of dyadic relationships using APIM (Byers & MacNeil, 2006; Sánchez-Fuentes & Santos-Iglesias), where despite observing interrelations between the variables of each couple member, it does not translate into partner effects. It is possible that these men are similar to their male partners in terms of the characteristics that contribute to sexual satisfaction, but the reciprocal association of these components may be more limited. Since there are very few works testing the model using APIM, future studies should implement this analytical strategy to observe whether this discrepancy is truly common in the IEMSS study framework.

In the case of female couples, in response to H1, as in men, the APIM showed three significant actor effects (GMREL, REW–CST, and CL_REW_–CL_CST_). Similar to what we found in male couples, no significant effects of the equality components (EQ_REW_ and EQ_CST_) were found in the APIM. This result slightly differed from those obtained in heterosexual couples by Sánchez-Fuentes and Santos-Iglesias (2016), who reported that equality of sexual costs (EQ_CST_) explained women’s own sexual satisfaction. In the case of our study, for women with same-sex partners, EQ_CST_ was associated with sexual satisfaction at the bivariate level. A similar finding was reported by Byers and Cohen (2017) in the validation of the IEMSS in women with same-sex partners, where EQ_REW_ was related to sexual satisfaction. Following Byers and MacNeil’s (2006) explanation, the absence in our APIM of effects of the equality components may be due to the considerably high sexual satisfaction of the couples in the present study, a finding that is consistent with previous evidence indicating that sexual satisfaction tends to be high especially for lesbians (Calvillo et al., 2020a, 2020b; Holmberg & Blair, 2009). Based on the above results, our first hypothesis is not fully supported as there is no association between all components of the IEMSS and sexual satisfaction in both male and female couples.

In line with previous evidence, in both male and female couples, the variable most strongly related to sexual satisfaction was GMREL. This association has been reported in several studies (Álvarez-Muelas et al., 2021; Byers, 2005; Calvillo et al., 2020a, 2020b; Lawrance & Byers, 1995; Mangas et al., 2024; McGuire & Barber, 2010; Sánchez-Fuentes & Santos-Iglesias, 2016) and does not seem to be associated with factors such as sex or sexual orientation (Kurdek, 2008).

On the other hand, regarding H2, two significant partner effects not present in male couples (i.e., negative effect of REW–CST and positive effect of CL_REW_–CL_CST_) were observed in female couples, indicating that the more female partners’ sexual rewards exceeded their sexual costs, the lower the sexual satisfaction experienced by them, and when the comparative level between perceived sexual rewards and costs exceeded that expected in their partners, the higher the sexual satisfaction reported by them. This confirms our second hypothesis about the presence of couple effects exclusively in the case of women, in line with previous evidence testing the IEMSS (Sánchez-Fuentes & Santos-Iglesias, 2016).

These partner effects, which are present in women but not in men, could be due to, as Sánchez-Fuentes and Santos-Iglesias (2016) noted, the fact that women, unlike men, are less focused on satisfying their own needs, paying more attention than men to the needs of their partners (Miller & Byers, 2004). In addition, lesbians tend to have high levels of emotional closeness (Spitalnick & McNair, 2005) and intimacy with their partners (Eldridge & Gilbert, 1990), paying special attention to the consensus or agreement that emerges among them (Mangas et al., 2024; Pérez-Amorós et al., 2024). Although women tend to emphasize the emotional aspects of sexual relationships more than men do in general (Peplau, 2003), those with same-sex partners perceive these emotional and relational aspects as rewarding to a greater extent (Cohen et al., 2008). In addition, female couples are characterized by high levels of communication about their sexual life (Jordan & Deluty, 2000), which strengthens the intimacy between them and contributes to better dyadic adjustment (Calvillo et al., 2020a, 2020b). Taken together, these findings highlight that in female couples, factors such as sexual intimacy, security, and emotional connection gain greater prominence and may promote more satisfying sexual experiences (Byers & Cohen, 2017; Scott et al., 2018).

Similarly to what occurred in heterosexual couples (Sánchez-Fuentes & Santos-Iglesias, 2016), women’s sexual satisfaction is impaired by a favorable balance of rewards and costs in their partner (REW–CST negative partner effect). That is to say, women’s sexual satisfaction—regardless of whether their partner is of different or the same sex—requires a balanced trade-off between the sexual rewards and costs of their partners. This effect may be due to socio-educational factors affecting the female population, such that women, regardless of their orientation, perceive a possible threat and a detriment to their sexual satisfaction when an imbalance exists with respect to their partner in terms of exchanges within their relationship. This effect may be useful for psychotherapists to pay attention to the degree of horizontality (i.e., understood as the degree of perceived equality/inequality between members of a couple, rejecting hierarchies) existing in female couples with respect to sexual rewards and costs. However, this negative REW–CST effect on sexual satisfaction differs from previous findings at the bivariate level, where the same variable was positively associated with sexual satisfaction. This discrepancy is also observed in previous evidence with Spanish heterosexual couples (Sánchez-Fuentes & Santos-Iglesias, 2016) and may be due to the greater complexity of the APIM analysis, an approach that should be implemented in the future to clarify the consistency of this particular result.

On the other hand, the positive partner effect of CL_REW_–CL_CST_, despite not having been found in the study by Sánchez-Fuentes and Santos-Iglesias (2016), appears reasonable given that women may experience greater sexual satisfaction when their partner realizes that the perceived reality of the relationship exceeds her own expectations. This fact may be associated with the strengths of same-sex couples, such as a greater ability to talk about one’s feelings (Riggle et al., 2016; Rostosky & Riggle, 2017), the degree to which they engage in intimate or conversational activities after sexual encounters, and the extent to which both consider each other’s desires, feelings, and needs (see Calvillo et al., 2024). This, in turn, could translate into them having more ability to inquire into what their partners interpret as a reward or a cost, and therefore, they would feel more satisfied if they are aware that their perceived exchanges exceed their partners’ expectations. Moreover, this fact could lead to an increase in their sexual self-esteem, a dimension that is also closely related to sexual satisfaction (Antičević et al., 2017; Peixoto et al., 2018). This significant association of partner effects in female couples could prove useful when in the context of sex or couples therapy. Especially in couples formed by two women, professionals should focus on the rewards and costs experienced by both partners due to the dyadic dynamics they could have both in themselves and in the other person.

The results obtained in this study with same-sex couples are very similar to those of Sánchez-Fuentes and Santos-Iglesias (2016) with heterosexual couples. Thus, we conclude that sexual satisfaction shows more similarities across sexual orientations than across sexes. This does not suggest that sexual orientation is not associated with the individual and dyadic experience of sexual satisfaction, but rather that the sex that a person has and the coincidence or not with that of the partner (i.e., different vs. same-sex couples) likely play a key role in both partners’ sexual satisfaction.

Due to the available evidence, we cannot assert that some of the components of the IEMSS perform better or are more relevant in male or female same-sex couples. Future research should aim to study the experiences of same-sex couples to clarify whether our findings are replicable. We consider it essential to further pay attention to the sex/gender configuration of couples in future dyadic studies, especially those using the IEMSS framework. The gender representation established by men and women can vary considerably and be influenced by a system of privileges and oppressions reflected in intimate relationships (Few-Demo & Allen, 2023). Lesbian relationships are often characterized by high emotional intensity, probably due to the “double dose” of gender socialization, which promotes closeness, intimacy, and communication (Riggle et al., 2016). However, men in same-sex relationships make greater efforts to maintain emotional boundaries and are less likely to consider sharing feelings as crucial (Umberson et al., 2015). In the words of Thomeer et al. (2020), neglecting gender dynamics in sexual and gender diversity couples “prevents a full understanding of gender within romantic partnerships within an increasingly sexual and gender diverse population” (p. 221).

This study has several limitations. Firstly, though the sample size exceeds the established thresholds for dyadic studies recommended by Kenny et al. (2006) and Fincham and Cui (2011), we cannot generalize the results to the Spanish population because of the cross-sectional design of the study and because participants were selected by non-probabilistic sampling. This approach does not ensure the statistical representativeness of the sample, which can introduce bias. Nevertheless, this method also makes it possible to collect valuable and relevant data that provide a solid basis for future research. Additionally, all participants were cisgender and binary, which excludes some other diversities, and most participants were relatively young, highly educated, and reported high levels of sexual and relationship satisfaction. Another factor that may have affected our results is the fact that male couples have a relatively longer relationship length than female couples. In addition, gender roles in Spain are currently changing, especially among young individuals, as Spain is a country with a high degree of acceptance and broad protection of the rights of LGBT+ people (according to ILGA World, 2020; Mendos et al., 2020). On the other hand, the battery of instruments was disseminated through social media, which makes it difficult for people without access to them to participate in the study. Finally, due to the scarcity of research on same-sex couples, and the absence of studies similar to the present one in terms of context, sexual identity, and analytical strategies, there is the limitation that many of our findings can be explained only in the context of other studies with heterosexual people. However, some works have shown that same-sex couples are also influenced by traditional gender roles, especially when they are victims of sexual prejudice (Napier et al., 2023) or when these roles affect the public sphere (Kowalski & Scheitle, 2020).

Despite these limitations, the results obtained are considered relevant from both clinical and research perspectives. Future works might consider incorporating experiences of trans, nonbinary, intersex, and LGBT+ people with functional and/or psychological diversities; serodiscordant couples; and people whose relationships fall under the umbrella of consensual non-monogamy. Given the nature of sexual satisfaction and the factors that influence it, dyadic factors that include both partners should be incorporated into future research on sexual satisfaction (Byers & Rehman, 2014; DeLamater & Hyde, 2004; Rehman et al., 2013). We believe that through this study, we moved our understanding forward, as we respond to the future lines of Byers and Cohen (2017), incorporating, in addition to the experiences of sexually diverse people, the dyadic approach for the first time, something that enriches the IEMSS literature. Moreover, as established by Byers and Cohen (2017), future work must examine the trajectory or stage of the couple relationship through longitudinal research. In this study, different stages of the relationship were analyzed together (e.g., dating and long-term relationships). Because the various stages of the relationship may affect experienced sexual satisfaction (Byers & Rehman, 2014), more research is needed to account for their specific effects, especially because recent studies suggest that the model may operate differently in younger and older adults (see Santos-Iglesias & Byers, 2021). Despite this, the IEMSS is effective in explaining sexual satisfaction independently of the effect of sociodemographic factors, including relationship length (Byers & MacNeil, 2006; Byers & Rehman, 2014; Lawrance & Byers, 1995). Although in this work we have used the original version of the IEMSS, the literature has been expanding its original components with relevant variables specific to the population under study. As such, future work could incorporate dimensions that affect sexual satisfaction of same-sex couples, such as internalized homonegativity (Bahamondes et al., 2023), body dissatisfaction (Sandoval et al., 2022), or communication skills (Newcomb et al., 2021), without forgetting variables such as minority stress caused by sexual prejudice, an aspect that may affect the experiences of this collective (Doyle & Barreto, 2023; Frost & LeBlanc, 2023; Frost et al., 2017). Regarding the components of the model, future work should further focus more on the equality components and evaluate same-sex couples whose partners are older (see Santos-Iglesias & Byers, 2021). Finally, future studies could also examine whether the current associations might be different for couples who show lower levels of sexual satisfaction or whose levels are discrepant between partners (e.g., using response surface analyses models).

### Implications and Conclusions

The obtained results have clinical implications for sexual satisfaction associated with dyadic factors (Byers & MacNeil, 2006; Byers & Wang, 2004; Purnine & Carey, 1997), especially for women (McClelland, 2011) and women belonging to sexual diversities (Byers & Cohen, 2017). Specifically, these findings provide psychotherapists with tools to improve couples’ sexual satisfaction, for example, by helping them optimize the quality of the non-sexual aspects of the relationship, to increase the level of sexual rewards and decrease the level of costs, to modify unrealistic sexual expectations that arise from comparing one’s own and others’ rewards and costs, and to develop an environment that results in more equal rewards and costs between partners, as already established by Byers (1999), among which we would like to add the possibility of evaluating the horizontality experienced by the couple in terms of sexual rewards and costs, as well as encouraging dialogue between partners on the exchanges that are considered rewarding or costly within the framework of the relationship. Although no partner effects were found in our study in male couples, we encourage further use of the dyadic approach in them as well (i.e., importance of assessing both partners). In line with previous research with the IEMSS as a reference, this study also emphasizes the importance of non-sexual factors in the relationship, as evidenced by the close link between sexual satisfaction and relationship satisfaction.

In conclusion, our results support the validity of the IEMSS (Lawrance & Byers, 1992, 1995) in understanding the sexual satisfaction of Spanish same-sex couples. Due to the similarity with the results obtained previously in different-sex couples, our findings suggest that sexual satisfaction and its manifestations could be more related to factors associated with gender than with the sexual orientation of the individuals. The IEMSS again proves to be an optimal tool for assessing sexual satisfaction, on this occasion in same-sex couples. To improve the sexual well-being and quality of life of this population, we recommend continuing to use this consolidated theoretical model, because, in the words of Byers and Cohen (2017), although sexual satisfaction takes center stage in many studies, some of which even use components of the model, most of the literature concerning the IEMSS has been developed by the same research teams. The IEMSS ensures robustness and consistency in analyzing the complex dimension of sexual satisfaction while providing a solid foundation that can adapt and evolve to address emerging challenges, ultimately enhancing our understanding and improvement of sexual well-being.

## Data Availability

The data presented in this study are available upon request to the authors.
